# Preoperative NT-proBNP and CRP predict perioperative major cardiovascular events in non-cardiac surgery

**DOI:** 10.1136/hrt.2009.181388

**Published:** 2009-10-26

**Authors:** J-H Choi, D K Cho, Y-B Song, J-Y Hahn, S Choi, H-C Gwon, D-K Kim, S H Lee, J K Oh, E-S Jeon

**Affiliations:** 1 Department of Medicine, Cardiovascular Imaging Center, Cardiac and Vascular Center, Samsung Medical Center, Sungkyunkwan University School of Medicine, Seoul, Korea; 2 Department of Emergency Medicine, Cardiovascular Imaging Center, Cardiac and Vascular Center, Samsung Medical Center, Sungkyunkwan University School of Medicine, Seoul, Korea; 3 Department of Cardiology, Hanmaeum General Hospital, Jeju, Korea; 4 Division of Cardiovascular Diseases, Mayo Clinic College of Medicine, Rochester, Minnesota, USA

## Abstract

**Objective:**

To investigate whether simple and non-invasive measurement of N-terminal pro-brain natriuretic peptide (NT-proBNP) and/or C-reactive protein (CRP) can predict perioperative major cardiovascular event (PMCE).

**Design:**

Prospective, single-centre, cohort study.

**Setting:**

A 1900-bed tertiary-care university hospital in Seoul, Korea

**Design and patients:**

The predictive power of NT-proBNP, CRP and Revised Cardiac Risk Index (RCRI) for the risk of PMCE (myocardial infarction, pulmonary oedema or cardiovascular death) were evaluated from a prospective cohort of 2054 elective major non-cardiac surgery patients. Optimal cut-off values were derived from receiver operating characteristic curve (ROC) analysis.

**Main outcome measurement:**

PMCE (myocardial infarction, pulmonary oedema or cardiovascular death) within postoperative 30 days.

**Results:**

PMCE developed in a total of 290 patients (14.1%). Each increasing quartile of NT-proBNP or CRP level was associated with a greater risk of PMCE after adjustment for traditional clinical risk factors. The relative risk (RR) of highest versus lowest quartile was 5.2 for NT-proBNP (p<0.001) and 3.7 for CRP (p<0.001). Both NT-proBNP (cut-off  = 301 ng/l) and CRP (cut-off  = 3.4 mg/l) predicted PMCE better than RCRI (cut-off  = 2) by ROC analysis (p<0.001). Moreover, the predictive power of RCRI (adjusted RR  = 1.5) could be improved significantly by addition of CRP and NT-proBNP to RCRI (adjusted RR 4.6) (p<0.001).

**Conclusions:**

High preoperative NT-proBNP or CRP is a strong and independent predictor of perioperative major cardiovascular event in non-cardiac surgery. The predictive power of current clinical risk evaluation system would be strengthened by these biomarkers.

Perioperative major cardiovascular events (PMCE) such as acute myocardial infarction, pulmonary oedema or primary cardiovascular death are important causes of morbidity in patients undergoing a major non-cardiac surgery.^1^ A number of clinical risk indices using scoring system have been developed, but the predictive power is still insufficient.^2^ ^3^ ^4^ ^5^ Moreover, the results of preoperative myocardial stress test were not consistently predictive of risk.^6^ ^7^ ^8^ A simple and strongly predictive non-invasive test is clinically warranted.

We hypothesised that the pathophysiology of cardiovascular disease including inflammation, myocardial ischaemia or increased ventricular filling pressures would be important in the development of PMCE. Then cardiovascular biomarkers reflecting this pathophysiology would be useful for the prediction of perioperative risk.^9^ Based on abundant clinical data, practical availability and background pathophysiology,^10^ ^11^ ^12^ we reasoned that N-terminal pro-brain natriuretic peptide (NT-proBNP) and C-reactive protein (CRP), representative biomarkers of haemodynamic stress and inflammation, respectively, would be predictive of PMCE. We investigated the predictive power of preoperative NT-proBNP and CRP and compared it with a well-validated clinical risk index for perioperative cardiovascular risk in a large prospective cohort of patients undergoing elective major non-cardiac surgery.

## Methods

### Patients

We enrolled patients who were referred to consulting cardiology physician for the evaluation of preoperative cardiovascular risk if the following criteria were fulfilled; (1) candidates for elective major non-cardiac surgery and aged more than 21 years, and (2) at least one of cardiovascular risk factors such as hypertension, diabetes, angina, history of revascularisation, heart failure or stroke, or (3) abnormal preoperative electrocardiography with pathological Q wave or non-sinus rhythm. Major non-cardiac surgery was defined by procedures performed in the operating room requiring general, spinal or epidural anaesthesia, after exclusion of very low-risk surgeries such as dermatological, ophthalmological, nasal or auditory procedures.

**Figure 1:**
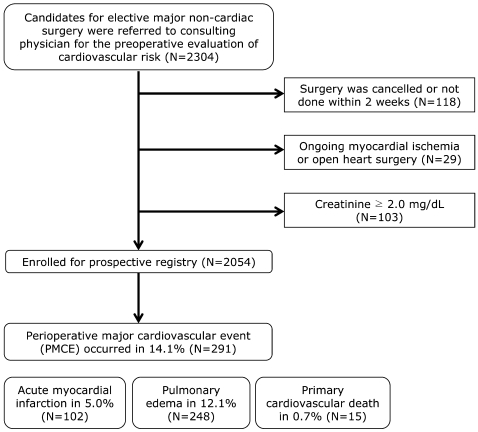
Study flowchart.

We prospectively enrolled 2304 consecutive patients from November 2004 to April 2008. The following patients were excluded; surgery was not done within 2 weeks (n = 118), significant myocardial ischaemia or those who required open heart surgery (n = 29). To avoid bias in the NT-proBNP results from renal insufficiency, 103 patients with preoperative serum creatinine ⩾2.0 mg/dl (⩾176.8 μmol/l) were also excluded.^13^ The remaining 2054 patients had undergone non-cardiac surgery within 2 weeks and constituted the study cohort (fig 1).

### Data collection

Clinical perioperative cardiovascular risk was assessed according to the Revised Cardiac Risk Index (RCRI) modified by Lee, a well-validated and widely used risk prediction index.^1^ ^4^ ^5^ ^8^ ^14^ Briefly, RCRI calculates perioperative risk by sum of points. Each risk factor, including high-risk surgical procedures, history of ischaemic heart disease, pulmonary oedema, cerebrovascular disease, insulin-dependent diabetes and serum creatinine >2.0 mg/dl, is assigned one point. The risk of major cardiac event including myocardial infarction, pulmonary oedema, primary cardiac arrest and complete heart block predicted by RCRI was known to be 0.4% to 11% according to an RCRI score of 0 to ⩾3.

The patient’s clinical history and functional capacity were evaluated according to the ACC/AHA guidelines on perioperative cardiovascular evaluation and care for non-cardiac surgery.^8^ Basic laboratory tests including electrocardiography, chest x-ray, NT-proBNP and CRP were evaluated within 2 weeks before surgery. Additional non-invasive tests were performed at the physician’s discretion. Electrocardiography and serum troponin I were evaluated at the end of the day of surgery and 24 hours later. A chest x-ray was taken on the next day. Any abnormal signs or symptoms suggesting pulmonary oedema or myocardial ischaemia were followed by meticulous evaluation of perioperative cardiac status with repeated cardiac serum markers and electrocardiography. If active pulmonary oedema or ongoing myocardial ischaemia was found, the patient was transferred to the cardiovascular team and treated appropriately. Patients were followed up by the consulting physician until discharge or up to 30 days in hospital after surgery. In case of mortality, the cause of death was decided on the consensus of the surgeon, anaesthesiologist and cardiovascular consulting physician.

The primary endpoint was a perioperative major cardiovascular event (PMCE), which was defined by any single or combined event of secondary endpoints including myocardial infarction, development of pulmonary oedema or primary cardiovascular death. Myocardial infarction was defined by a rise in postoperative troponin I above the 99th percentile of the upper reference limit (0.78 ng/ml, Roche Diagnostics, Switzerland). Diagnosis of pulmonary oedema required a formal reading of chest x-ray by a radiologist consistent with the complication. Primary cardiovascular death was defined by sudden death that could not be explained by any other non-cardiovascular postoperative complications. All clinical events were collected by a research nurse and investigated by physicians. This study protocol was approved by the institutional review board of Samsung Medical Center.

### Statistics

Perioperative risk predictors (RCRI, CRP and NT-proBNP) were treated as continuous variables or ordered categorical variables. Logarithmically transformed values of NT-proBNP and CRP were also used to minimise distribution skewness and kurtosis. The dose-response relation between risk predictors and clinical outcome was investigated by the Jonckheere-Terpstra test for trend. The predictive power of each risk predictor was also quantitatively evaluated with relative risk per 1 SD increase of RCRI and logarithmically transformed biomarker levels. Receiver-operating characteristic (ROC) analysis was performed to calculate sensitivity, specificity, area under the curve and the optimal cut-off. The predictive power of each method was compared using the Hanley and McNail method.^15^ Relative risk was calculated by Zhang and Yu’s method.^16^ Relative risk was also calculated for the risk predictors with values categorised by optimal cut-off levels, or a combination of these cut-off levels in an additive manner. Independent predictors of PMCE in univariate analysis were included in multivariate logistic models with forward conditional methods. A p value <0.05 (two-sided) was considered significant. SPSS version 13.0 was used mostly. ROC curves were compared using Medcalc version 9.6.

## Results

### Clinical characteristics

Preoperative clinical characteristics of the study population are shown in table 1. Briefly, most patients had good functional status without overt heart failure (functional class I or II in 94.6% and no heart failure in 97.0%). A history of angina was found in 13.2%, including 8.0% of myocardial infarction. Percutaneous coronary intervention (PCI) or coronary artery bypass surgery (CABG) had been performed before non-cardiac surgery in 14.8%. Two-dimensional echocardiography was performed in most patients (93.6%), revealing abnormal left ventricular wall motion in 19.9% of patients. Evidence of myocardial ischaemia which was defined by positive non-invasive test or significant coronary artery stenosis was found in 21.6%. A preoperative β-blocker or statin was used in 17.3% and 14.8%, respectively.

**Table 1:** Baseline clinical characteristics

	Frequency (%) or median (interquartile range)
Age (years)	68 (61–73)
Male gender	1247 (60.7)
Functional class III or IV	112 (5.5)
Diabetes	355 (17.3)
Diabetes treated with insulin	71 (3.5)
Hypertension	1247 (60.7)
Previous or current heart failure	62 (3.0)
Previous stroke	188 (9.3)
Angina	270 (13.2)
Previous myocardial infarction	165 (8.0)
Previous revascularisation*	304 (14.8)
Creatinine (mg/dl)	0.9 (0.7–1.1)
NT-proBNP (ng/l)	109.3 (47.0–352.8)
C-reactive protein (mg/l)	2.0 (0.7–8.0)
Electrocardiography	2054 (100)
Pathological Q waves	111 (5.4)
Atrial fibrillation	164 (8.0)
Left bundle branch block	14 (0.7)
Echocardiography	1923 (93.6)
Left ventricular ejection fraction ⩽40%	95 (4.6)
Abnormal left ventricular wall motion	408 (19.9)
Preoperative non-invasive test†	765 (37.2)
Overall positive result for ischaemia	188 (9.2)
Preoperative Invasive test	544 (26.5)
Significant coronary artery disease by invasive test	359 (17.5)
Any evidence of myocardial ischaemia§	444 (21.6)

Data are shown as frequency (%) or median (interquartile range) as appropriate.

*Previous revascularisation includes percutaneous coronary intervention in 222 cases and bypass surgery in 82 cases.

†Preoperative non-invasive test includes SPECT in 577 cases, Treadmill test in 113 cases and stress echocardiography in 47 cases.

§Positive non-invasive test or coronary artery stenosis of more than 50% was defined as any evidence of myocardial ischaemia.

### Perioperative clinical evaluation

Most patients received general anaesthesia (97.1%). Ninety-three patients (4.5%) underwent urgent surgery within 24 hours after consultation because of altered clinical situation. These cases were not excluded from the analysis (table 2).

**Table 2:** Surgical procedure and perioperative risk

	Frequency (%)
Vascular surgery	531 (25.9)
Aorta	160 (7.8)
Suprainguinal vascular	98 (4.8)
Infrainguinal vascular	158 (7.7)
Carotid endarterectomy	97 (4.7)
Other vascular	18 (0.9)
Non-vascular surgery	1520 (74.4)
Thorax	85 (4.1)
Abdomen	501 (24.4)
Head and neck	178 (8.7)
Orthopaedic	439 (21.4)
Prostate	82 (4.0)
Neurosurgery	55 (2.7)
Other surgery	182 (8.9)
General anaesthesia	1994 (97.1)
Urgent surgery	93 (4.5)
RCRI (median, interquartile range)	1 (0–2)
RCRI = 0	555 (27.0)
RCRI = 1	846 (41.2)
RCRI = 2	579 (28.2)
RCRI = 3	69 (3.4)
RCRI = 4	5 (0.2)
High risk surgery by RCRI*	844 (41.1)

Data are shown as frequency (%).

*Defined by intraperitoneal, intrathoracic or suprainguinal vascular surgery according to RCRI (Revised Cardiac Risk Index modified by Lee).

### Clinical outcomes

Perioperative major cardiovascular event (PMCE) had developed in 290 (14.1%) patients. Individual patients may have had more than one event, and all events were counted as an incidence. There were 102 (5.0%) acute myocardial infarction, 248 (12.1%) pulmonary oedema and 20 (1.0%) deaths, which were similar to previous studies evaluated patients with risk of cardiovascular disease.^1^ Five patients died because of postoperative disease progression or surgical complication (0.2%), and 15 deaths were determined to be primary cardiovascular death (0.7%), which included three (0.2%) acute myocardial infarction, two (0.1%) stress-induced cardiomyopathy,^17^ four (0.2%) aortic aneurysm rupture or dissection, one (0.1%) stroke and five (0.2%) sudden death of unknown cause (fig 1) (table 3).

**Table 3:** Clinical outcome

	Frequency (%)
Perioperative major cardiovascular event (PMCE)	290 (14.1)
Acute myocardial infarction	102 (5.0)
Revascularisation	26 (1.3)
Percutaneous coronary intervention	24 (1.2)
Coronary artery bypass surgery	2 (0.1)
New or aggravated heart failure	248 (12.1)
Primary cardiovascular death*	15 (0.7)
Acute myocardial infarction	3 (0.2)
Stress induced cardiomyopathy	2 (0.1)
Aortic disease	4 (0.2)
Stroke	1 (0.1)
Unknown	5 (0.2)
Death due to postoperative complication or disease progression	5 (0.2)
All death	20 (1.0)

Data are shown as frequency (%).

*Death that was not caused by postoperative complication or underlying non-cardiovascular disease progression.

### Predictive power of perioperative risk predictors

**Figure 2:**
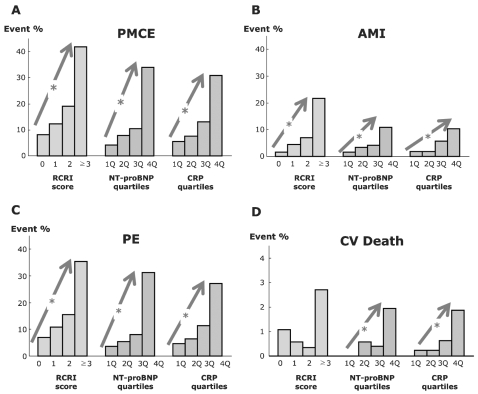
Clinical outcomes according to the risk predictors. AMI, acute myocardial infarction; CV death, primary cardiovascular death; PMCE, perioperative major cardiovascular event; PE, pulmonary oedema. *p<0.001 by Jonckheere-Terpstra test for trend.

We evaluated the continuous values of three risk predictors against perioperative events. Not only increasing score of a clinical predictor, RCRI, but also increasing quartile of biomarker levels, NT-proBNP and CRP, was associated with a greater risk of PMCE (p<0.001) (fig 2).

Next we investigated whether the risk predictors are related to clinical outcomes independently each other. Each 1-SD increase in RCRI (1 to 2) (adjusted relative risk (RR)  = 1.26 (95% CI 1.10 to 1.44)), log CRP (2.7 to 15.1 mg/l) (1.74 (1.55 to 1.95)) or log NT-proBNP (135 to 601 ng/l) (2.17 (1.91 to 2.45)) was associated with 1.3-fold to 2.2-fold increased rate of PMCE, even after adjustment for other risk predictors and traditional clinical risk factors. By subgroup analysis, CRP and NT-proBNP were also significantly associated with all secondary endpoints, whereas RCRI was not associated with AMI or primary cardiovascular death. Each 1-SD increase in log CRP or log NT-proBNP was also associated with 1.6-fold to 2.3-fold increased risk of PMCE (table 4).

**Table 4:** Clinical outcomes according to the risk predictors

	PMCE		AMI		PE		CV death	
	RR (95% CI)	p Value	RR (95% CI)	p Value	RR (95% CI)	p Value	RR (95% CI)	p Value
Per 1-SD increase*								
RCRI	1.26 (1.10 to 1.44)	0.001	1.18 (0.88 to 1.56)	0.27	1.30 (1.13 to 1.49)	<0.001	0.53 (0.29 to 1.00)	0.05
CRP	1.74 (1.55 to 1.95)	<0.001	1.58 (1.28 to 1.93)	<0.001	1.86 (1.64 to 2.10)	<0.001	2.16 (1.32 to 3.51)	0.002
NT-proBNP	2.17 (1.91 to 2.45)	<0.001	1.55 (1.28 to 1.88)	<0.001	2.27 (1.97 to 2.62)	<0.001	2.30 (1.48 to 3.56)	<0.001
**Optimal cut-off of each risk predictors**†								
RCRI	1.50 (1.17 to 1.91)	0.002	1.14 (0.70 to 1.86)	0.59	1.52 (1.17 to 1.96)	0.002	0.38 (0.08 to 1.71)	0.21
CRP	2.75 (2.16 to 3.45)	<0.001	2.62 (1.66 to 4.08)	<0.001	2.97 (2.28 to 3.81)	<0.001	5.38 (1.50 to 18.78)	0.010
NT-proBNP	3.89 (3.15 to 4.74)	<0.001	2.54 (1.68 to 3.79)	<0.001	4.72 (3.72 to 5.89)	<0.001	5.39 (1.86 to 15.30)	0.002
**Combination of best cut-off of each predictors§**								
RCRI or CRP or NT-proBNP⩾ cut-off§	4.55 (3.69 to 5.52)	<0.001	3.24 (2.10 to 4.92)	<0.001	5.64 (4.49 to 6.96)	<0.001	7.71 (2.48 to 23.32)	<0.001

Investigate independent association of each risk predictors with clinical outcomes were shown as adjusted relative risk (RR) with 95% confidence intervals (CI), all three risk predictors were included in the logistic regression analysis with forward conditional method. Analysis was adjusted with all significant univariate risk factors including age and sex.

*Measured in linear values, 1-SD increase from mean corresponded to 1 to 2 for RCRI, 135 ng/l to 601 ng/l for NT-proBNP, and 2.7 mg/l to 15.1 mg/l for CRP, respectively.

†Optimal cut-off values were ⩾2 for RCRI, ⩾301 ng/l for NT-proBNP, and ⩾3.4 mg/l for CRP.

§Defined by at least two of three risk predictors are higher than cut-off values.

AMI, acute myocardial infarction; CV Death, primary cardiovascular death; PE, pulmonary oedema; PMCE, perioperative major cardiovascular event.

### Augmentation of predictive power of clinical risk index by addition of biomarkers

Risk predictors categorised by optimal cut-off levels were used to test whether the predictive power could be increased by the combination of multiple risk factors. RCRI cut-off (⩾2) was associated with 1.5-fold increased risk of PMCE after adjustment for age, sex, and traditional clinical risk factors (adjusted RR  = 1.50 (95% CI 1.17 to 1.91)). The risk of PMCE based on CRP cut-off (⩾3.4 mg/l) and NT-proBNP cut-off (⩾301 ng/l) were much higher, 2.7-fold and 3.9-fold, respectively (CRP cut-off, 2.75 (2.16 to 3.45); NT-proBNP cut-off, 3.89 (3.15 to 4.14)) (table 4). Higher CRP and NT-proBNP values were also associated with 2.5-fold to 5.4-fold increased risk of secondary endpoints including AMI, pulmonary oedema, and primary cardiovascular death (p<0.05), whereas higher RCRI was not associated with AMI or primary cardiovascular death (table 4).

**Figure 3:**
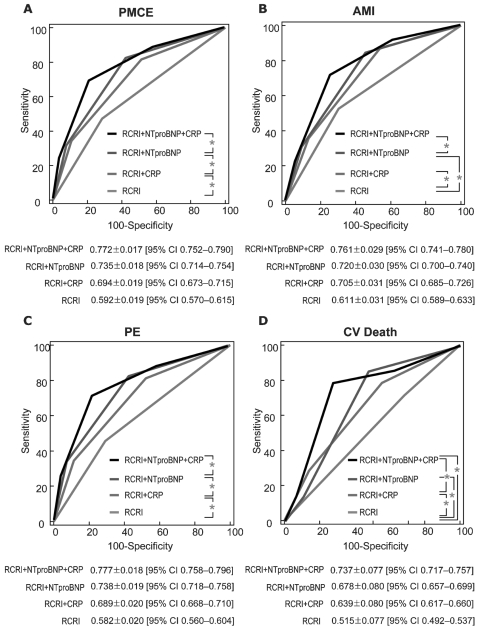
Receiver-operating characteristic (ROC) analysis of perioperative risk predictors. The predictive power of each combination of categorised risk predictor in an additive manner was investigated. Risk predictors were categorised according to the optimal cut-off levels derived from ROC analysis, which were 2 for RCRI, 301 ng/l for BNP and 3.4 mg/l for CRP. Areas under the curve (AUCs) with 95% CI are shown below each panel. *p<0.05 by Hanley and McNail methods. (A) ROC for PMCE. *p<0.001 for all, except RCRI + NT-proBNP vs RCRI + NT-proBNP + CRP (p = 0.001), and RCRI + CRP vs RCRI + NT-proBNP (p = 0.010). (B) ROC for AMI. *p<0.001 for all, except RCRI + NT-proBNP vs RCRI + NT-proBNP + CRP (p = 0.026), RCRI + CRP vs RCRI + NT-proBNP + CRP (p = 0.002) and RCRI + CRP vs RCRI + NT-proBNP (p = 0.590). (C) ROC for pulmonary oedema. *p<0.001 for all, except RCRI + CRP vs RCRI + NT-proBNP (p = 0.004) and RCRI + NT-proBNP vs RCRI + NT-proBNP + CRP (p = 0.001). (D) ROC for primary cardiovascular death. *RCRI vs CRP, p = 0.021; RCRI vs RCRI + NT-proBNP, p = 0.012; RCRI vs RCRI + NT-proBNP + CRP, p = 0.002; RCRI + CRP vs RCRI + NT-proBNP + CRP, p = 0.021. AMI, acute myocardial infarction; CV death, primary cardiovascular death; PE, pulmonary oedema; PMCE, perioperative major cardiovascular event.

The addition of biomarkers to RCRI increased the relative risk of RCRI for clinical events threefold. For PMCE, the adjusted RR of RCRI increased from 1.50 (95% CI 1.17 to 1.91) to 4.55 (3.69 to 5.52) after addition of CRP and NT-proBNP. The relative risks for secondary endpoints including AMI, pulmonary oedema and primary cardiovascular death also increased threefold to sevenfold (table 4). The increase of predictive power of RCRI by addition of biomarkers to RCRI was again calculated using ROC analysis. For PMCE, AUC (area under curve) of combination of RCRI cut-off (⩾2) and NT-proBNP cut-off (⩾301 ng/l), 0.735 (0.714 to 0.754), and AUC of combination of RCRI cut-off and CRP cut-off (⩾3.4 mg/l), 0.694 (0.673 to 0.715), were significantly higher than AUC of RCRI cut-off, 0.592 (0.570 to 0.615), (p<0.001, each). AUC of combination of RCRI cut-off and NT-proBNP cut-off and CRP cut-off, 0.772 (0.752 to 0.790), was even much higher than AUC of RCRI cut-off (p<0.001) (fig 3A). The increase in AUC by addition of biomarker cut-offs to RCRI cut-off was also evident in all secondary endpoints including AMI, pulmonary oedema and primary cardiovascular death (fig 3B-D).

## Discussion

Our study showed that the predictive power of a current perioperative clinical risk index could be strengthened significantly by the simple addition of the cardiovascular biomarkers, NT-proBNP and CRP. Our findings can be summarised that the average sensitivity of predicting perioperative major cardiovascular event increased from 59% to 77% after addition of biomarkers to clinical risk prediction system.

### Predictive power of biomarker versus clinical risk index

The Revised Cardiac Risk Index modified by Lee (RCRI), which has been shown to be superior to other perioperative risk indices was selected as the clinical risk predictor in our study.^2^ ^3^ ^4^ ^5^ The results showed that a single cardiovascular biomarker, NT-proBNP or CRP, is superior to clinical risk indices for the prediction of perioperative events. In addition, both NT-proBNP and CRP were significantly associated not only with all clinical events but also with all subsidiary events including AMI, pulmonary oedema and primary cardiovascular death, whereas RCRI was not associated with primary cardiovascular death. Furthermore, NT-proBNP and CRP were not only useful for risk prediction but also shown to improve the predictive power of clinical risk index. The addition of NT-proBNP and CRP to the clinical risk index increased the adjusted relative risk threefold.

### Role of NT-proBNP and CRP in the pathophysiology of perioperative cardiovascular events

The pathophysiology of perioperative myocardial infarction has been explained by responses to perioperative surgical stress represented by a catecholamine surge with associated haemodynamic stress, systemic inflammation and hypercoagulability.^18^ ^19^ High NT-proBNP was associated not only with a high risk of pulmonary oedema but also with AMI and primary cardiovascular death in our study. This could be explained by the release of NT-proBNP or B-natriuretic peptide (BNP) from subclinical ischaemic or injured myocardial tissue regardless of haemodynamic stress.^12^ ^20^ Our results strongly suggest that NT-proBNP could be marker of myocardial ischaemia or generalised cardiac impairment in perioperative situations as well as non-surgical situations. The predictive power of CRP was also better than the clinical risk index in this study. However, the high CRP levels did not predict clinical events beyond NT-proBNP. Previous population-based studies showed that the value of CRP in cardiovascular risk prediction was moderate or less than traditional risk factors.^21^ ^22^ A recently published study showed that NT-proBNP was better than CRP for the prediction of sudden cardiac death.^23^ A relatively high CRP level in perioperative conditions might lead to a greater contribution of CRP to the risk prediction in our study.

### Limitations

This study was performed at single centre. Only patients who had undergone formal preoperative cardiovascular consultation were included. However, given the strength of our results and wide variability in the predictive power of risk indices which highly depend on study population, it is unlikely that enrolment of more patients would have changed the main results of our study.^1^ Long-term follow-up after discharge was not performed, although most postoperative cardiovascular events are known to develop in early postoperative periods.^24^ ^25^ Exclusion of patients with renal dysfunction might have affected the predictive power of RCRI, which includes renal function evaluation.^13^ ^26^ Therefore, our results cannot be generalised to patients with renal dysfunction. The presence of preoperative infection or the use of antibiotics, which might affect the level of CRP or the severity of perioperative systemic inflammation, was not used as an exclusion criterion. The relation between infection and perioperative cardiovascular disease is little known and requires further investigation. The use of a preoperative β-blocker or statin was less than 20% and was not associated with clinical events in our study. The benefit of a β-blocker or statin is currently in debate even in high-risk patients, and large prospective trials are needed to confirm it.^6^ ^27^ ^28^

In conclusion, our results demonstrate that high preoperative NT-proBNP and CRP levels are strong and independent predictors of perioperative cardiovascular events following non-cardiac surgery. Furthermore, the predictive power of the current clinical perioperative risk index could be strengthened significantly by the addition of these biomarkers. Evaluation of preoperative NT-proBNP and CRP is a practical, simple and reasonable strategy to improve perioperative risk prediction with minimal clinical burden and cost.
